# Health disparities among highly vulnerable populations in the United States: a call to action for medical and oral health care

**DOI:** 10.3402/meo.v18i0.20644

**Published:** 2013-03-26

**Authors:** Allison A. Vanderbilt, Kim T. Isringhausen, Lynn M. VanderWielen, Marcie S. Wright, Lyubov D. Slashcheva, Molly A. Madden

**Affiliations:** 1 Center on Health Disparities, School of Medicine, Virginia Commonwealth University, Richmond, VA, USA; 2 Department of Oral Health Promotion and Community Outreach, School of Dentistry, Virginia Commonwealth University, Richmond, VA, USA; 3 Department of Health Administration, School of Allied Health, Virginia Commonwealth University, Richmond, VA, USA; 4 School of Dentistry, Virginia Commonwealth University, Richmond, VA, USA

**Keywords:** interprofessional education, underserved populations, vulnerable populations, health disparities, oral health, medical health

## Abstract

Healthcare in the United States (US) is burdened with enormous healthcare disparities associated with a variety of factors including insurance status, income, and race. Highly vulnerable populations, classified as those with complex medical problems and/or social needs, are one of the fastest growing segments within the US. Over a decade ago, the US Surgeon General publically challenged the nation to realize the importance of oral health and its relationship to general health and well-being, yet oral health disparities continue to plague the US healthcare system. Interprofessional education and teamwork has been demonstrated to improve patient outcomes and provide benefits to participating health professionals. We propose the implementation of interprofessional education and teamwork as a solution to meet the increasing oral and systemic healthcare demands of highly vulnerable US populations.

Healthcare in the United States (US) is burdened with enormous healthcare disparities associated with a variety of factors including insurance status, income, and race (1). Highly vulnerable populations, classified as those with complex medical problems exacerbated by social needs (2), are one of the fastest growing segments within the US (3). Highly vulnerable individuals include racial and ethnic minorities who have complex chronic illnesses and multiple chronic comorbidities. Between 2000 and 2010, the prevalence of chronic disease comorbidities has significantly increased for Hispanic (32.2–42.4%) and black (43.8–51.6%) populations over the age of 65, in addition to those 65+ living below 100% of the federal poverty level (FPL) (42.5–50.0%) and between 100 and 199% of the FPL (41.4–49.5%) (3). To address the complex needs of these patient populations, healthcare practitioners must understand social determinants of health and utilize a comprehensive health definition, including biological, social, and psychological dimensions (4).

Over a decade ago, the US Surgeon General publically challenged the nation to realize the importance of oral health and its relationship to general health and well-being (5), yet oral health disparities continue to plague the US healthcare system (6, 7). Over 45 million adults and children reside in dental professional shortage areas (8), and more than half of uninsured low-income children have not had preventative dental care visits (9, 10). In addition, low-income adults are highly unlikely to have dental checkups (11). Poor dental health increases the risk for diabetes, heart disease, premature birth, and poor birth outcomes (12). Associations have been documented between periodontal disease and diabetes (13), cardiovascular disease (14), and gastrointestinal (GI) disorders (15). Additionally, there is an increased risk for developing gingivitis and potentially periodontal disease, among older adults who may be diabetic and/or taking certain anti-hypertension drugs (16). Aged individuals, as well as those taking numerous medications, may experience *xerostomia*, a condition that predisposes them to various oral infections and other adverse oral conditions (17) such as tooth decay. New and innovative strategies are needed to meet the ‘Triple Aim’ of oral health to improve patient experience, population health, and efficiency (18). One innovative response to these emerging findings is to shift toward an interdisciplinary model of care, incorporating dental professionals as a core part of the team (19–21).

In the US, health care professionals are overwhelmingly trained in uniprofessional settings, independent of interprofessional education (IPE) and collaboration, leading to challenges in practice (22). Such teams have been shown to reduce medical errors and improve health outcomes among patients with chronic conditions (23). Interprofessional experiences also benefit healthcare professionals by improving interprofessional competence, described as one's knowledge of other professionals including an understanding of their training and skillsets, and role clarity (24) – two key components of interprofessional teamwork. In fact, over a decade ago, the Institute of Medicine (IOM) initiated the call to action among healthcare students and professionals to collaborate on interdisciplinary teams and engage in quality improvement, yet dental care providers are still often overlooked when defining such teams (25).

The interprofessional concept in the education of health professionals (23) and oral health professionals is essential to closing the gap within the US for the poor and highly vulnerable populations. IPE has the potential to foster the necessary collaboration, communication, and teamwork to provide the comprehensive health care required to meet the oral and systemic health challenges of the 21st century, and it creates a collaborative decision-making environment to provide optimal patient care (26). Since the IOM's initial call for IPE over a decade ago, researchers have demonstrated that collaborative interprofessional practice can play a significant role in mitigating health disparities within the US and around the world (26).

We challenge schools of medicine, dentistry, pharmacy, nursing, allied health, public health, and social work to collaborate when developing curriculum and to build a sustainable foundation for a systematic approach to IPE and collaboration with the focus on reducing oral and systemic health disparities within the US. This team-based approach has the potential to improve patient experience and population health and optimize efficiency. It is time to take action and demand implementation of IPE and collaborative care in order to decrease health disparities, improve health outcomes for the poor and highly vulnerable, and train future health care professionals with the skills necessary for the 21st century and beyond.
